# Nutritional Properties of Milk from Dairy Ewes Fed with a Diet Containing Grape Pomace

**DOI:** 10.3390/foods11131878

**Published:** 2022-06-24

**Authors:** Francesca Bennato, Andrea Ianni, Marco Florio, Lisa Grotta, Francesco Pomilio, Maria Antonietta Saletti, Giuseppe Martino

**Affiliations:** 1 Faculty of BioScience and Technology for Food, Agriculture, and Environment, University of Teramo, 64100 Teramo, Italy; fbennato@unite.it (F.B.); aianni@unite.it (A.I.); mflorio@unite.it (M.F.); lgrotta@unite.it (L.G.); 2 Istituto Zooprofilattico Sperimentale Dell’Abruzzo e del Molise “G. Caporale”, 64100 Teramo, Italy; f.pomilio@izs.it (F.P.); m.saletti@izs.it (M.A.S.)

**Keywords:** grape pomace, ewes, milk, fatty acids, caseins

## Abstract

The aim of the present study was to evaluate the effects of a diet containing a 10% of grape pomace (GP) on the milk yield, chemical-nutritional characteristics, total phenolic compounds (TPCs), antioxidant activity (AOA), fatty acids and proteins profile of dairy ewe’s milk. Forty-six ewes were dived into two groups: a control group (Ctrl), fed a standard diet, and an experimental group (GP+), whose diet was supplemented with 10% of GP on dry matter. The trial lasted 60 days and milk samples were collected and analyzed at the beginning (T0) and after 60 (T60) days. Dietary enrichment with GP did not affect the yield and the chemical composition of the milk. TPCs and AOA were not affected by the diet. After 60 days, the diet induced an increase in monounsaturated fatty acids (MUFA) and a decrease in medium chain saturated fatty acids (MCSFA), but the total saturated fatty acids (SFA), polyunsaturated fatty acids (PUFA), short chain saturated fatty acids (SCSFA) and long chain saturated fatty acids (LCSFA) were not modified. A decrease in the C14 desaturation index and an increase in the C18 index were also detected. Total caseins and whey protein were not affected by GP, even if a lower content of k-casein in GP+ milk compared to Ctrl milk was observed on the 60th day. The results of the present study suggest that 10% of GP can be included in the diet of lactating ewes without modifying milk gross composition but inducing significantly changes the fatty acid profile.

## 1. Introduction

The use of agri-food by-products in animal nutrition represents a fairly consolidated strategy that has its foundations in two main aspects. First of all, the valorization of these by-products represents a sustainable method both from an environmental and an economic point of view, since the industries that accumulate these wastes incur costs for their disposal. In addition to this, it should be emphasized that these matrices are rich in compounds with high biological value, and this generally has positive effects on the qualitative traits of products of animal origin, as well as on the animal welfare [1,2,3]. For instance, it has been demonstrated that the use of olive crude phenolic concentrate obtained from olive oil wastewater in the diet of dairy ewes induced an increase in milk polyunsaturated fatty acids (PUFA) [4]. The inclusion of pomegranate pulp in the diet of sheep reduced the concentration of saturated fatty acids (SFA) and increased that of PUFA [5]. In addition, in the milk of cows fed a diet containing dried grape pomace, there has been observed a significant increase in lactose and β-lactoglobulin but not in α-lactalbumin, albumin and caseins [6].

However, the utilization of agro-industry by-products in animal feeding has limits due to the presence of antinutritional factors such as lectin, tannin and non-starch polysaccharide, which can influence feed palatability, digestibility, and animal performance [7,8].

One of the by-products that has attracted more attention is certainly grape pomace, derived from grape processing in the wine industry, a particularly developed sector in Europe, with Italy and France representing the major producers [9]. This waste is characterized by the richness of tannins, phenolic compounds credited with antioxidant and anti-inflammatory functions and by the presence of PUFA, the consumption of which, as known, is associated with a lower risk of chronic diseases of the cardiovascular system [3,10].

The introduction of grape pomace in the diet of dairy ruminants has shown several advantages, particularly related to the fatty acids profile of milk and cheeses. Most of the studies were specifically conducted on dairy cows, evidencing the ability of this ingredient to influence rumen function, slowing down the last steps of biohydrogenation, thus favoring the accumulation of trans-vaccenic acid (C18:1, t11) [11]. This compound represents the precursor of rumenic acid (C18:2, c9, t11), a conjugate of linoleic acid (CLA) to which is associated high bioactive potential [12]. With particular regard to cattle, the introduction of grape pomace in their diet was also effective in reducing the production of ruminal methane, especially by inducing changes in the rumen microbiota [13]. With specific regard to the ruminal function, various considerations have also been reported for small ruminants [14], even with regard to the mechanisms associated with the biohydrogenation process [15].

There is still little knowledge on the dietary effect of grape pomace on the quality of ewes’ milk and deriving dairy products. Conversely to reports for dairy cows [3], the marked ability of this ingredient to modify some parameters of interest such as, for example, the fatty acids profile of ewes’ milk, has not been highlighted [16]. Therefore, the aim of the present study was to evaluate if a diet containing 10% of grape pomace (GP) was able to induce changes in milk yield, chemical-nutritional characteristics, total phenolic compounds (TPCs), antioxidant activity (AOA), fatty acids and proteins profile of dairy ewes milk.

## 2. Materials and Methods

### 2.1. Experimental Design and Animal Management

The animals were managed according to Directive 2010/63/EU of the European Parliament (European Union, 2010) and Directive 86/609/EEC (European Economic Community, 1986), which deals with the protection of animals used for experimentation or other scientific purposes [17,18].

Forty-six crossbreed dairy ewes were involved in this study. All the animals were in the first part of the lactation (<45 days in milk) and, as a result, were homogeneous for milk production, body weight, and number of lactations. Ewes were randomly assigned to two groups of 23 animals each and confined into two separated areas: a control group (Ctrl) and an experimental group (GP+). The trial lasted for 70 days, preceded by 10 days adaptation period to the experimental diet. During the adaptation period, in both groups, the concentrate was gradually increased up to 1 kg of DM. This was achieved on day 10, which was considered time zero. Animals received, for 60 days, alfalfa hay ab libitum and a custom-formulated concentrate, the ingredients and chemical composition of which is reported in Table 1. The concentrate ration (1 kg/day per head) was administered in correlation with two daily milkings (8:00 and 18:00).

The control group (Ctrl) received a standard diet according to the common nutritional needs of dairy ewes in lactation [19], while, in the experimental group (GP+), a 10% (*w*/*w*) of grape pomace (on a dry matter basis) was used as a substitute for beet pulp. Specifically, red pomace, derived from the processing of grapes obtained from vines of Montepulciano d’Abruzzo (Abruzzo Region, Italy), was used; grape pomace before its use was treated as previously described by Ianni et al. [20]. Briefly, GP was subjected to a fermentation phase and treatment with steam to eliminate ethyl alcohol and, subsequently, it was treated with water at 90 °C in order to recover the tartaric acid. Finally, the GP was dried for the production of flour, incorporated in concentrate formulation.

### 2.2. Sampling of Feed and Milk

The feeds of each group were collected at the beginning and at the end of the trial. The total amount collected at each time and for each group was mixed, and a final sample was taken. Samples of feed were taken and analyzed for chemical composition.

At the end of the adaptation period and on 60th day, the daily milk yield, determined as the sum of morning and evening milkings, was measured by using lactometers and milk samples of the morning milking were collected separately from each ewe. Fresh milk was immediately used for the chemical-centesimal analysis and the remaining part was suitably aliquoted and frozen at −20 °C before carrying out further analysis.

### 2.3. Chemical Analysis of Feed and Milk

The samples of concentrate were analyzed for dry matter (method 930.15), crude protein (method 954.01), ether extract (method 920.39), crude fiber (method 962.09) and ash (method 942.05) according to AOAC International [21]. Milk fat, protein, casein, lactose, and urea contents were determined using a MilkoScan FT 6000 (Foss Integrator IMT: Foss Analytics, Hiller’ød, Denmark); pH values were determined by using a portable pH meter equipped with an electrode (Crison, Barcelona, Spain).

### 2.4. Feed and Milk Phenolic Content and Antioxidant Capacity

The content of TPCs in feed and milk was determined according to the protocol reported by Singleton and Rossi [22]. Both in the feeds and in the defatted milk, the phenolic compounds were previously extracted by a hydroalcoholic solution (ethanol:water 70:30, *v*/*v*). The samples were agitated for 40 min in the dark, centrifugated and the supernatants were recovered, filtered and analyzed. The content of TPCs was determined according to Ianni et al. [23]. Briefly, 200 μL of extract was mixed with 1 mL of 0.2 N Folin–Ciocalteu reagent and 800 μL of sodium carbonate 7.5% in water. The samples were incubated at room temperature (20–22 °C) in the dark and after 30 min the absorbance at 765 nm (Jenway 6305 UV/Vis spectrophotometer, Jenway, Barloworld Scientific, Dunmow, United Kingdom) was evaluated. The results were reported as mg of gallic acid equivalent (GAE)/g of sample on a dry matter basis for the feeds and as μg GAE/mL for the milk. The AOA was determined as reported by Ianni et al. [23], mixing 100 μL of extract with 1 mL of opportunely diluted ABTS solution. The colorimetric evaluations were performed at 734 nm after 4 min and the results were expressed as μmol equivalent antioxidant capacity (TEAC)/g of sample on a dry matter basis for the feeds and as μg GAE/mL for the milk. 

### 2.5. Milk Protein Extraction and SDS-PAGE

Fifteen mL of each milk sample were cool centrifugated at 4000× *g* rpm for 15 min. The fat was removed, the supernatant was recovered, filtered and quantified using Bradford method [24] and Bovine Serum Albumine (BSA) as standard. Protein samples (7.5 μg) were diluted in sample buffer (0.5 M Tris-HCl, pH 6.8; 2% (*w*/*v*) SDS; 7% (*v*/*v*) glycerol; 4.3% (*v*/*v*) β-mercaptoethanol; 0.0025% (*w*/*v*) bromophenol blue), boiled for 5 min and loaded into a 12% polyacrylamide gel. After the run, the gels were placed for 30 min in a staining solution containing 40% (*v*/*v*) methanol, 10% (*v*/*v*) acetic acid and 0.1% Comassie Brillant Blue G-250. Thereafter, gels were de-stained by two washings in distilled water containing 40% (*v*/*v*) methanol and 10% (*v*/*v*) acetic acid. The densitometric values of caseins and whey proteins were analyzed by using ImageLab 6.0.1 software.

### 2.6. Feed and Milk Fatty Acid Profile

Feed fatty acids were extracted using a mix of chloroform and methanol (2:1, vol/vol) and analyzed by a gas chromatography (GC) coupled with a flame ionization detector (FID) equipped with a capillary column (Restek rt-2560 Column, fused silica 100 m × 0.25 mm highly polar phase; Restek Corporation, Bellefonte, PA, USA). Hydrogen was used as a carrier gas a flow rate of 1 mL/min. For the separation of methyl esters of fatty acids (FAMEs) the temperature program previously reported by Bennato et al. was used [25]. The milk lipid fraction was analyzed according to the AOAC official method [26]. The detection of FAMEs was performed by GC-FID. The injector temperature was 280 °C. The oven temperature was initially settled at 80° C and held for 10 min, then increased up to 172 °C at 4 °C/min for 30 min, finally increased up to 190 °C at 4 °C/min and held for 10 min. The FAMEs were identified by a comparison of the FAME retention times with different FAME analytical standards for feed (FAME Mix C8-C24 Supelco, Bellefonte, PA, USA) and milk (FIM-FAME-7-Mix; Matreya LLC, Pleasant Gap, PA, USA). Peak areas were quantified by Chrome Card software and the relative value of each individual fatty acid was expressed as a relative percentage of the total FAMEs identified. The relative percentage of each fatty acid was also used to calculate the sum of SFA, MUFA = monounsaturated fatty acids (MUFA), PUFA, short chain saturated fatty acids (SCSFA), medium chain saturated fatty acids (MCSFA) and long chain saturated fatty acids (LCSFA). Atherogenic Index (AI), Thrombogenic Index (TI) and Desaturation (C14, C16, C18 and CLA) Indices were calculated as previously reported by Innosa et al. [27].

### 2.7. Statistical Analysis

Statistical data analysis was performed using the JMP Pro 14 program (SAS Institute, Cary, NC, USA). All data were processed with ANOVA (Analysis of Variance) to analyze the impact of the diet and time. Sample means were assessed by HSD Tukey’s test and differences were considered significant for *p* < 0.05. Results were reported as means ± standard error (SE). 

## 3. Results

### 3.1. Diet Fatty Acid Profile, Polyphenol Content and Antioxidant Activity 

The fatty acid composition of the diet administrated to the animals is reported in Table 2. In both groups, the most abundant fatty acid was linoleic acid (C18:2, c9, c12). Oleic acid (C18:1, c9) and palmitic acid (C16:0) were also presented, whereas stearic (C18:0) and linolenic (C18:3, c9, c12, c15) acids were the fatty acids less present. No significant differences in TPCs and AOA were found between the two diets. 

### 3.2. Milk Yield, Chemical Composition, Polyphenols Content and Antioxidant Capacity

The average daily milk yield at the beginning of the study was 1.58 ± 0.09 kg/head and 1.62 ± 0.11 for Ctrl and GP+, respectively. After 60 days of experimental period, the daily milk yield decreased significantly, both in Ctrl (*p* < 0.05) and GP+ (*p* < 0.001), reaching values of 1.28 ± 0.07 and 1.18 ± 0.10 Ctrl and GP+, respectively. Moreover, there was no significant difference in milk yield between Ctrl and GP+, either at the beginning or after 60 days.

As reported in Table 3, during the entire period of the trial, the chemical composition of the milk samples was not affected by diet, with the exception of lactose, whose values were lower (*p* < 0.01) in GP+ compared to Ctrl on the 60th day. In Ctrl, the milk lactose percentage increased significantly at 60 days compared to T0; conversely, in GP+, the content remained constant throughout the entire period. Both in Ctrl and GP+, the lipid percentage increased significantly during the trial and significant differences were observed in both groups between the beginning and after 60 days of experiment. In both groups, urea content showed a decrease after 60 days. No significant differences within treatments and between treatments were observed in casein and protein content and pH values. 

**Figure 1:**
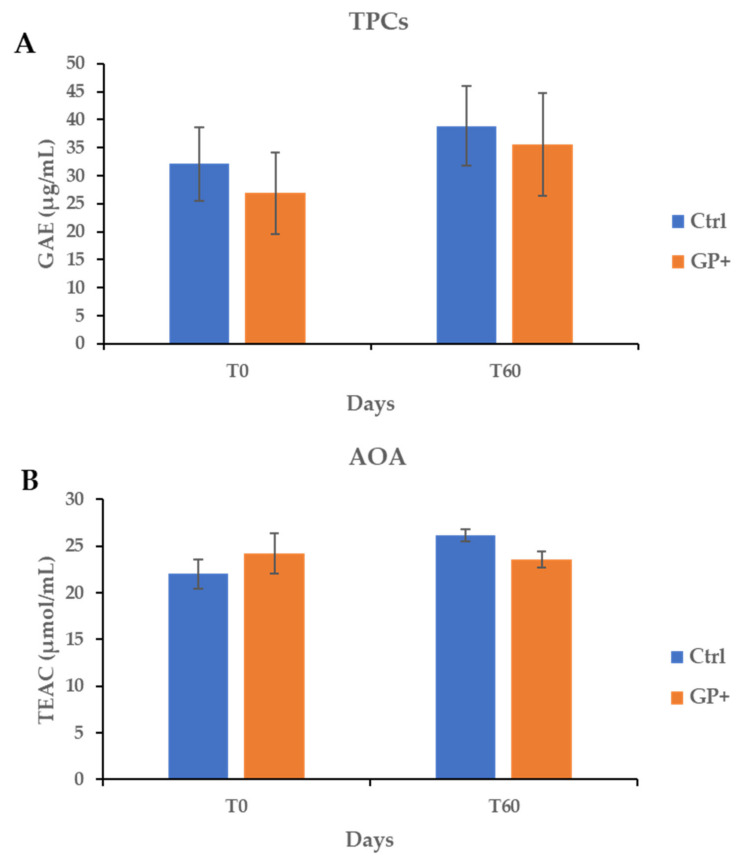
Total Phenolic Compounds (TPCs) (**A**) and Antioxidant Activity (AOA) (**B**) at the beginning (T0) and after 60 (T60) days of trial in raw milk samples obtained from ewes fed a standard diet (Ctrl) and grape pomace diet (GP+). GAE = gallic acid equivalent; TEAC = trolox equivalent antioxidant capacity. Data are reported as mean ± SE for n = 23. Differences between Ctrl and GP+ were not significant (*p* > 0.05).

The TPCs and AOA in ewes’ milk were not affected by the diet (Figure 1). No significant differences in TPCs and AOA both in Ctrl and GP+ were observed during the trial.

### 3.3. SDS-PAGE Milk Protein Profile

**Figure 2:**
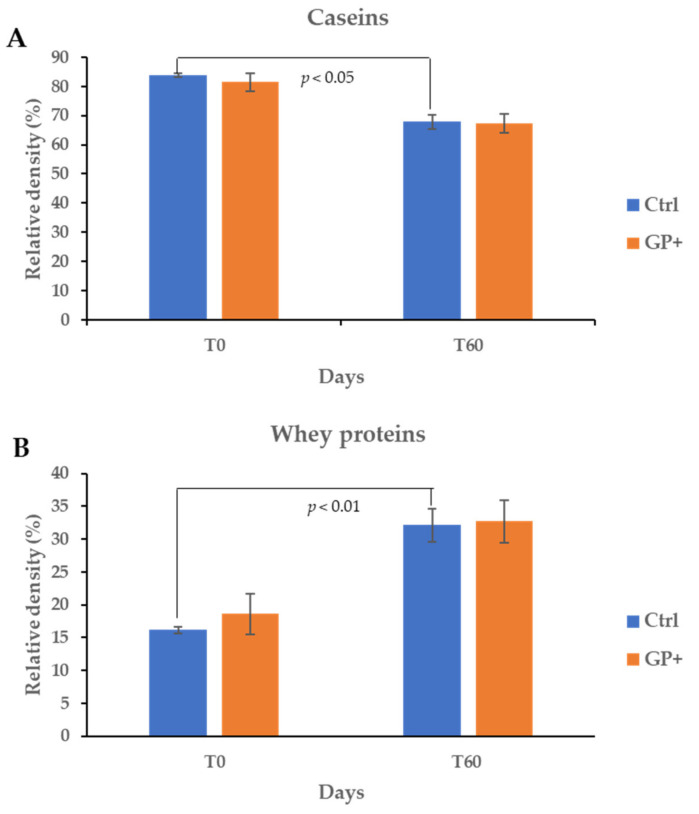
Total caseins (**A**) and whey proteins (**B**) at the beginning (T0) and after 60 (T60) days of trial in raw milk samples obtained from ewes fed a standard diet (Ctrl) and grape pomace diet (GP+). Data are reported as mean percentage ± SE for n = 23. Differences between Ctrl and GP+ were not significant (*p* > 0.05).

The sodium dodecyl sulfate polyacrylamide gel electrophoresis (SDS-PAGE) allowed us to separate the major proteins in ewes milk (α-casein, β-casein, k-casein, β-lactoglobulin and α-lactalbumin). Attention was paid particularly to the determination of the total caseins (α-casein, β-casein, k-casein) and whey proteins (β-lactoglobulin and α-lactalbumin). As reported in Figure 2, no significant differences between the two groups were found both in caseins and whey proteins at the beginning and after 60 days. However, in Ctrl milk, lower (*p* < 0.05) levels of caseins (Figure 2A) and higher (*p* < 0.01) whey proteins (Figure 2B) content were found at 60 days compared to T0 samples. Conversely, in GP milk, no significant differences in caseins and whey proteins content between T0 and T60 were detected.

Specifically, the main changes, both in Ctrl and GP+ proteins profile, were relative to a significant decrease in β-casein between T0 and T60 (Table 4). Conversely, after 60 days, the values of k-casein and β-lactoglobulin increased, both in Ctrl and Gp+ milk. At 60 days, the values of k-casein in GP+ milk were significantly lower (*p* < 0.05) compared to Ctrl. No significant differences between Ctrl and GP+ were detected in all the other protein fractions at T0 and T60.

### 3.4. Milk Fatty Acids Profile

The composition of milk fatty acids in Ctrl and GP+ at the beginning and at the end of the study is shown in Table 5. The inclusion of GP modified the milk fatty acids profile. After 60 days, significantly higher values of MUFA (*p* < 0.05) and lower levels of MCSFA (*p* < 0.01) were detected in GP+ milk compared to the Ctrl, but total SFA, PUFA, SCSFA and LCSFA content was not modified. In addition, at the end of the trial compared to the beginning of the experimental period, GP+ milk samples contained a lower percentage of C15:0 (*p* < 0.01) and C14:1, c9 (*p* < 0.001). After 60 days, a lower content of C12:0 (*p* < 0.05), C14:0 (*p* < 0.05) and C14:1, c9 (*p* < 0.01) was highlighted in GP+ milk samples compared to Ctrl. A lower (*p* < 0.05) C14 desaturation index and higher C18 desaturation index were found in T60 GP+ samples. No significant differences in C16 and CLA desaturation indices and TI and AI were found between the two groups.

## 4. Discussion

The results of the present study revealed that GP supplementation in the diet of lactating ewes did not influence milk yield during the whole study. Both Ctrl and GP+ follow the same trend characterized by a decrease in milk yield. This trend follows the general pattern of the lactation curve in dairy sheep, characterized by an increase from lambing to the peak of lactation and a gradual decrease toward the end of lactation with different persistence, depending on rearing conditions, breed, and individual animal [28,29]. Similarly, studies conducted on dairy cows fed a diet containing GP showed no significant effects on milk yield [3,30].

The stage of lactation, as well as genic and physiological factors, breed, milking interval and techniques, breeding strategies and nutrition, affect milk composition [29,30,31,32]. Ewes milk is characterized by a low quantity of fat and proteins at the beginning of the lactation and highest content at the end of lactation stage [33]. It is widely reported that a negative correlation between milk yield and milk composition at a higher milk production is usually associated with a lower fat and protein concentration [34,35]. In our study, lipid content increased in both groups on the 60th day. Conversely, protein content showed higher stability, probably reflecting the lower variation in protein content throughout the lactation. As reported in other studies, the use of GP in the diet did not affect fat and protein concentration [16]. Conversely to a report by Manso et al. [16], the dietary inclusion with GP did not affect lactose percentage during the period; in fact, in GP+, the values of lactose remained constant up to sixty days. On the contrary, on the 60th day, in Ctrl, the amount of lactose increased. This finding disagrees with data in literature that reported a low content of lactose in sheep milk as in other ruminants at the beginning of lactation toward the end of lactation, contrary to the behavior of fat and protein contents in the milk [36,37]. The mechanism governing this process is very difficult to understand and little is known about its variation in ewes. It is known that lactose synthesis is predominantly subjected to hormonal control with very few dietary factors influencing its production [38,39,40]. However, the significant increase in lactose in Ctrl milk was not associated with an increase in milk volume production, of which the lactose is the major determinant, since the entry of water into the vesicle formed in the trans-Golgi is linked with lactose synthesis to maintain osmotic equilibrium with surrounding fluids. Thus, the rate of lactose synthesis regulates water secretion and, consequently, milk yield. Lactose is the major sugar of milk, and the glucose is the primary substrate for its synthesis. Lactose synthesis is initiated in the Golgi apparatus and continues in the vesicles with an influx of water and ionic constituents that causes the vesicles to swell as they pass toward the cell surface. Glucose and uridine diphosphate (UDP)-galactose combine to form lactose under the action of the enzyme lactose synthetase. The milk protein α-lactalbumin must be present for glucose and UDP-galactose to combine. Thus, α-lactalbumin appears to be a prime regulator of lactose synthesis [41,42].

However, contrary to what is expected, electrophoretic profile analysis did not show a significant increase in α-lactalbumin in Ctrl. GP supplementation did not affect the α-lactalbumin quantity in accordance with a study by Chedea et al. [6] in dairy cows fed a diet containing 15% GP. Conversely to a report by Chedea and colleagues [6], in our study, GP did not affect β-lactoglobulin percentage. Concerning the caseins fractions, GP did not modify α-casein and β-casein amount, but a significant increase in k-casein was observed, even if the percentage of k-casein in GP+ was, however, lower compared to Ctrl on 60 days.

Caseins are the main protein in sheep milk, and they are responsible for the enzymatic coagulation of milk and their hydrolysis pattern is an important characteristic of cheese ripening, so they are positively correlated with cheese production [43]. The different protein profile found in the milk of the two groups may induce changes in the cheese-making property of the milk, influencing the quality and the yield of the cheeses. 

The amount of phenolic compound in the milk depends mainly on the type of diet. In our experiment, the diets administered to animals were characterized by similar polyphenols concentrations, so the TPCs and AOA in ewes’ milk did not differ between the two groups.

The fatty acids composition of the milk varies according to the stage of lactation, which can influence the milk fatty acids profile as result of the balance between body fat mobilization and de novo synthesis in the mammary gland. However, feeds composition and feeding strategies have a decisive influence on the milk composition, especially fat LCSFA. In the milk of ewes fed GP, lower values of C15:0 and C14:1, c9 were highlighted in T60 samples compared to T0 milk. After 60 days of 10% GP inclusion in the diet, GP+ milk compared to Ctrl had a lower percentage of MCSFA and lower levels of C12:0, C14:0 and C15:0. A decrease of C10:0 and C12:0 was also observed in Churra ewes fed a diet containing 10% of GP [11] and in dairy cows [13]. Saturated fatty acids from C6:0 to C14:0 are almost exclusively synthesized ex novo by the mammary gland, and it has been demonstrated that their synthesis is related to the dietary intake of fermentable carbohydrate [44]. As reported by those authors, the high amount of lignin in GP, whose rumen digestion is limited, may interfere with de novo synthesis of fatty acids in the mammary gland. In our experiment, dietary supplementation with GP resulted in increased MUFA concentrations in the milk, suggesting that ruminal biohydrogenation was indeed inhibited.

Saturated fat intake is usually associated with an increased risk of coronary artery disease or cardiovascular disease. However, studies on the effects of C12:0 and C14:0 on human health are often contradictory. Lauric (C12:0), myristic (C14:0) and palmitic (C16:0) acids seem to exert atherogenic effects by increasing the synthesis of (low-density lipoprotein) LDL cholesterol and the level of total cholesterol [45] and the potential of C14:0 to increase total serum cholesterol would seem fourfold or even sixfold higher than that of C16:0. However, several studies have demonstrated that lauric acid increases the level of high-density lipoprotein (HDL) [45]. The low amount of MCFA, as C12:0 and C14:0, detected in GP+ milk may represent a benefit for the consumer.

In order to better evaluate the nutritional and health aspects of the milk, IA and TI were also calculated. These indices are used to value the nutritional and animal fat for consumers. In this study, we did not find differences related to the diet in AI and TI, whose values remained constant during the period in both groups. To study the effect of the diet on the capacity of stearoyl-CoA desaturase (SCD) to desaturate SFA to Δ^9^-unsaturated fatty acids, the desaturation indices for C16:1, c9 C18:1, c9, C14:1, c9, and CLA were the estimated [15]. On the 60th day, the lower C18 index found in Ctrl compared to T0 samples, suggests that a low quantity of the C18:0 that is transferred from the blood to the mammary gland is desaturated in the mammary gland to produce C18:1, c9. This trend was not observed in GP+, in which a higher value was detected at 60 days. This finding suggests that milk fluidity was maintained, since the conversion of 18:0, absorbed by the mammary gland to C18:1, c9 is essential to maintain the fluidity of the milk. Conversely, to 60 days, the higher C14:1, c9 level in GP+ milk compared to T0 milk, might be the result of an increase in the desaturation of C14:0 occurring in the mammary gland by SCD, a finding also supported by the higher values of the relative C14 index. The only source of C14:1, c9 in the milk, is the desaturation of C14:0; fatty acid was produced almost entirely in the mammary gland, so the C14 index is the main representative of the SCD compared to the other indices [46]. In cow milk somatic cells, a positive correlation between C14 index and SCD mRNA has been observed [47]. The lower C14 index in GP+ milk could be due to the stimulatory effect of GP compounds on the expression, or on the activity of SCD.

## 5. Conclusions

The results of the present study suggest that grape pomace can be included in the diet of lactating ewes at a level of 10% without adverse effects on milk production and chemical composition. The different protein profile found in the milk of ewes fed grape pomace may affect the cheese-making property of the milk, influencing the quality and the yield of the cheeses, an aspect that needs further investigation. In addition, grape pomace is able to modify the milk fatty acid profile, whose composition is one of the factors that affects the formation and the development of cheese aroma. The low content of saturated fatty acids, such as lauric and myristic in the milk of ewes fed with grape pomace, also represents a benefit for human health.

## Figures and Tables

**Table 1:** Nutrient and chemical composition of the diet administered to the control group (Ctrl) and experimental group (GP+).

	Ctrl	GP+
**Ingredients (%)**		
Triticum	20.20	19.70
Corn	18.00	19.00
Barley	14.00	15.00
Sunflower	10.00	7.00
Beet pulps	10.00	-
Grape Pomace	-	10.00
Soy	10.00	11.50
Wheat	8.00	8.00
White sorghum	5.00	5.00
Calcium carbonate	1.50	1.50
Sodium bicarbonate	1.00	1.00
Molasses	1.00	1.00
Dicalcium phosphate	0.80	0.80
Vitamins and microelements	0.50	0.50
**Chemical composition (%)**		
Dry Matter	89.00	88.00
Crude protein ^1^	18.76	19.09
Ether extract ^1^	3.03	3.30
Crude fiber ^1^	6.97	7.27
Ash ^1^	6.63	6.70
ME ^1^, MJ/kg	7.67	7.35

ME = Metabolizable Energy. ^1^ Data are reported on dry matter basis.

**Table 2:** Fatty acid profile, total phenolic content and antioxidant activity of the diet administered to the control group (Ctrl) and experimental group (GP+).

	Ctrl	GP+
**Fatty acids ^1^ (%)**		
C14:0	0.35 ± 0.19	0.19 ± 0.01
C16:0	16.84 ± 1.67	16.67 ± 1.41
C18:0	1.81 ± 0.05	1.73 ± 0.25
C16:1, c9	0.17 ± 0.03	0.16 ± 0.03
C18:1, c9	18.94 ± 0.65	18.68 ± 0.31
C18:1, c11	0.18 ± 0.09	0.20 ± 0.06
C18:2, c9, c12	56.56 ± 1.59	57.75 ± 1.53
C18:3, c9, c12, c15	0.48 ± 0.09	0.40 ± 0.02
C22:0	0.06 ± 0.01	0.05 ± 0.01
C22:1	0.12 ± 0.05	0.11 ± 0.05
Others	4.49 ± 0.20	4.13 ± 0.27
SFA	19.06 ± 1.79	18.64 ± 1.18
MUFA	19.41 ± 0.75	18.71 ± 1.02
PUFA	57.04 ± 0.20	58.14 ± 1.53
TPCs ^2^ (mg GAE/g)	2.19 ± 0.07	2.24 ± 0.13
AOA ^2^ (μmol TEAC/g)	461.90 ± 31.49	502.59 ± 29.20

SFA = Saturated Fatty Acid, MUFA = Monounsaturated Fatty Acid, PUFA = Polyunsaturated Fatty Acid, TPCs = Total Phenolic Compounds, AOA = Antioxidant Activity. ^1^ Data are reported as mean relative percentage of total FAMEs ± SE. ^2^ Data are reported on dry matter basis. Differences in TPCs, AOA and fatty acid profile between Ctrl and GP+ were not significant (*p* > 0.05).

**Table 3:** Chemical composition of at the beginning (T0) and after 60 (T60) days of trial of raw milk samples obtained from ewes fed a standard diet (Ctrl) and grape pomace diet (GP+).

	T0			T60		
	Ctrl (n = 23)	GP+ (n =23)	*p*	Ctrl (n = 23)	GP+ (n = 23)	*p*
**Lipid (%)**	5.29 ± 0.25 ^b^	5.04 ± 0.22 ^b^	ns	6.38 ± 0.27 ^a^	6.12 ± 0.21 ^a^	ns
**Casein (%)**	4.48 ± 0.09	4.47 ± 0.10	ns	4.18 ± 0.08	4.35 ± 0.08	ns
**Urea (mg/dL)**	66.40 ± 1.38 ^a^	65.42 ± 1.40 ^a^	ns	58.53 ± 1.42 ^b^	58.10 ± 1.33 ^b^	ns
**Lactose (%)**	5.04 ± 0.05 ^b^	5.03 ± 0.05	ns	5.24 ± 0.06 ^a^	4.98 ± 0.06	<0.01
**Protein (%)**	5.68 ± 0.10	5.82 ± 0.09	ns	5.43 ± 0.09	5.56 ± 0.10	ns
**pH**	6.69 ± 0.20	6.75 ± 0.20	ns	6.85 ± 0.25	6.85 ± 0.30	ns

Data are reported as mean ± SE. ^a,b^ Means in the same row with different uppercase superscript letters are significantly different by time (*p* < 0.05). ns = not significant (*p* > 0.05).

**Table 4:** Densitometric analysis of SDS-PAGE protein bands at the beginning (T0) and after 60 (T60) days of trial in raw milk samples obtained from ewes fed a standard diet (Ctrl) and grape pomace diet (GP+).

	T0			T60		
	Ctrl (n = 23)	GP+ (n = 23)	*p*	Ctrl (n = 23)	GP+ (n = 23)	*p*
**α-casein (%)**	12.43 ± 0.91	12.79 ± 2.35	ns	8.01 ± 1.10	9.60 ± 0.69	ns
**β-casein (%)**	61.85 ± 2.52 ^a^	60.61 ± 2.26 ^a^	ns	37.77 ± 2.36 ^b^	43.27 ± 2.60 ^b^	ns
**κ-casein (%)**	9.56 ± 0.62 ^b^	8.01 ± 1.67 ^b^	ns	22.13 ± 1.54 ^a^	14.45 ± 1.43 ^a^	<0.05
**β-lactoglobulin (%)**	10.12 ± 0.77 ^b^	11.82 ± 2.12 ^b^	ns	22.23 ± 2.26 ^a^	23.92 ± 2.30 ^a^	ns
**α-lactalbumin (%)**	6.04 ± 0.35	6.74 ± 1.14	ns	8.89 ± 0.92	8.77 ± 4.66	ns

Data are reported as mean percentage ± SE of the main proteins found in the electrophoretic profile of each sample. ^a,b^ Means in the same row with different uppercase superscript letters are significantly different by time (*p* < 0.05). ns = not significant (*p* > 0.05).

**Table 5:** Fatty acid profile at the beginning (T0) and after 60 (T60) days of trial in raw milk samples obtained from ewes fed a standard diet (Ctrl) and grape pomace diet (GP+).

	T0			T60		
	Ctrl (n = 23)	GP+ (n = 23)	*p*	Ctrl (n = 23)	GP+ (n = 23)	*p*
**Fatty Acids (%)**						
C4:0	2.69 ± 0.37	2.60 ± 0.33	ns	3.13 ± 0.20	3.41 ± 0.28	ns
C6:0	3.04 ± 0.35	2.85 ± 0.33	ns	3.31 ± 0.15	3.24 ± 0.24	ns
C8:0	3.63 ± 0.33	3.39 ± 0.35	ns	3.73 ± 0.10	3.38 ± 0.24	ns
C10:0	13.14 ± 0.86	12.12 ± 0.95	ns	13.20 ± 0.27	11.26 ± 0.64	ns
C12:0	7.98 ± 0.36	7.17 ± 0.39	ns	7.24 ± 0.26	6.12 ± 0.34	<0.05
C14:0	13.17 ± 0.28	13.14 ± 0.34	ns	14.21 ± 0.27	13.38 ± 0.49	<0.05
C15:0	1.12 ± 0.05	1.13 ± 0.04 ^a^	ns	1.08 ± 0.02	0.92 ± 0.04 ^b^	<0.01
C16:0	26.22 ± 0.86	27.69 ± 0.95	ns	26.47 ± 0.60	27.89 ± 1.19	ns
C17:0	0.44 ± 0.03	0.50 ± 0.04	ns	0.46 ± 0.02	0.42 ± 0.03	ns
C18:0	5.50 ± 0.43	6.19 ± 0.42	ns	6.23 ± 0.24	6.06 ± 0.63	ns
C14:1, c9	0.44 ± 0.02	0.48 ± 0.02 ^a^	ns	0.51 ± 0.02	0.38 ± 0.02 ^b^	<0.001
C16:1, c9	1.21 ± 0.05	1.27 ± 0.05	ns	1.22 ± 0.04	1.38 ± 0.10	ns
C18:1, t11	1.01 ± 0.60	0.42 ± 0.08	ns	0.45 ± 0.05	1.76 ± 0.92	ns
C18:1, c9	11.69 ± 0.77	12.79 ± 0.92	ns	11.34 ± 0.49	12.30 ± 0.79	ns
C18:1, c11	0.13 ± 0.03	0.10 ± 0.01	ns	0.08 ± 0.01	0.09 ± 0.02	ns
CLA	1.13 ± 0.08	1.18 ± 0.10	ns	1.10 ± 0.05	1.20 ± 0.11	ns
C 18:2, c9, c11	3.37 ± 0.44	2.97 ± 0.21	ns	2.42 ± 0.14	3.23 ± 0.46	ns
C 18:3, c9, c12, c15	0.77 ± 0.06	0.79 ± 0.06	ns	0.57 ± 0.03	0.55 ± 0.05	ns
Others	3.31 ± 0.08	3.22 ± 0.05	ns	3.26 ± 0.08	3.02 ± 0.11	ns
SFA	76.94 ± 1.26	76.78 ± 1.23	ns	79.06 ± 0.75	76.09 ± 1.44	ns
MUFA	14.48 ± 0.79	15.06 ± 0.91	ns	13.59 ± 0.55	15.91 ± 0.93	<0.05
PUFA	5.27 ± 0.53	4.93 ± 0.35	ns	4.09 ± 0.53	4.98 ± 1.74	ns
SCSFA	5.73 ± 0.72	5.45 ± 0.66	ns	6.43 ± 0.34	6.65 ± 0.49	ns
MCSFA	39.05 ± 1.59	36.96 ± 1.79	ns	39.45 ± 0.64	35.06 ± 1.38	<0.01
LCSFA	32.16 ± 1.26	34.37 ± 1.30	ns	33.17 ± 0.52	34.37 ± 1.35	ns
C14 Index	32.38 ± 0.17	31.81 ± 0.19	ns	35.48 ± 0.17	28.17 ± 0.24	<0.05
C16 Index	44.56 ± 0.21	44.01 ± 0.13	ns	44.30 ± 0.16	46.62 ± 0.19	ns
C18 Index	68.24 ± 0.66 ^a^	67.32 ± 0.63	ns	64.45 ± 0.56 ^b^	67.71 ± 1.41	<0.05
CLA Index	68.49 ± 0.59	74.46 ± 3.67	ns	71.83 ± 2.63	63.21 ± 7.82	ns
AI	4.64 ± 0.37	4.59 ± 0.33	ns	5.25 ± 0.31	4.45 ± 0.44	ns
TI	8.30 ± 0.36	8.04 ± 0.08	ns	7.53 ± 0.15	7.92 ± 0.29	ns

Data are reported as mean relative percentage of total FAMEs ± SE. CLA = Conjugated Linoleic Acids; SFA = Saturated Fatty Acids; MUFA = Monounsaturated Fatty Acids; PUFA = Polyunsaturated Fatty Acids; SCSFA = Short Chain Saturated Fatty Acids (C4:0-C6:0), MCSFA = Medium Chain Saturated Fatty Acids (C8:0-C15:0); LCSFA = Long Chain Saturated Fatty Acids (C16:0-C18:0); C14 Index = [(C14:1, c9)/(C14:0 + C14:1, c9)]*100; C16 Index = [(C16:1, c9)/(C16:0 + C16:1, c9)]*100; C18 Index = [(C18:1, c9)/(C18:0 + C18:1, c9)]*100; CLA Index = (CLA/(C18:1, t11 + CLA)]*100; AI = Atherogenic Index; TI = Thrombogenic Index. ^a,b^ Means in the same row with different uppercase superscript letters are significantly different by time (*p* < 0.05). ns = not significant (*p* > 0.05).

## Data Availability

The data presented in this study are available on request from the corresponding author.
